# MicroRNAs as Novel Biomarkers for Breast Cancer

**DOI:** 10.1155/2010/950201

**Published:** 2009-07-20

**Authors:** H. M. Heneghan, N. Miller, A. J. Lowery, K. J. Sweeney, M. J. Kerin

**Affiliations:** Department of Surgery, Clinical Science Institute, National University of Ireland, Galway, Ireland

## Abstract

Breast cancer is a complex phenotypically diverse genetic disease, involving a variety of changes in gene expression and structure. Recent advances in molecular profiling technology have made great progress in unravelling the molecular taxonomy of breast cancer, which has shed new light on the aetiology of the disease and also heralded great potential for the development of novel biomarkers and therapeutic targets. Mi(cro)RNAs are a contemporary class of small noncoding endogenous RNA molecules, generating great excitement in the clinical and scientific communities. The recent discovery that miRNA expression is frequently dysregulated in cancer has uncovered an entirely new repertoire of molecular factors upstream of gene expression, which warrants extensive investigation to further elucidate their precise role in malignancy. We present a comprehensive and timely review of the role of miRNAs in cancer: addressing miRNA function, their putative role as oncogenes or tumor suppressors, with a particular emphasis on breast cancer throughout. We discuss the recent discovery of quantifiable circulating cancer-associated miRNAs, which heralds immense potential for their use as novel minimally invasive biomarkers for breast and other cancers. Finally, we comment on the potential role of miRNAs in breast cancer management, particularly in improving current prognostic tools and achieving the goal of individualized cancer treatment.

## 1. Introduction

The molecular biology of malignancy is diverse, complex, and remains poorly understood. The incidence of malignancies such as breast cancer is increasing consistently, and breast cancer has now become the commonest form of female malignancy among women in almost all of Europe and North America. Each year more than 1.3 million women will be diagnosed with breast cancer worldwide and approximately 4652 000 will die from the disease [1] despite the fact that breast cancer is highly curable if diagnosed and treated appropriately at an early stage. In Ireland alone, the annual incidence is currently over 2300 and rising [2]. The value of current histological prognostic indicators in predicting the course of the disease is weak and many of the molecular mechanisms underlying breast cancer progression remain poorly understood. This deficit has led to significant interest in the quest for novel predictive markers for breast cancer. 

**Figure 1:**
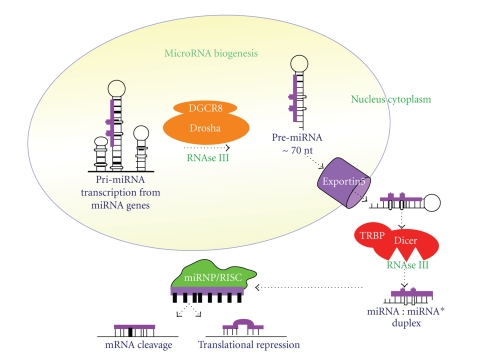
MiRNA biogenesis and processing in human cells: the multistep process begins in the nucleus where the RNase III enzyme Drosha, coupled with its binding partner DGCR8, cleaves nascent miRNA transcripts (pri-miRNA) into ~70 nucleotide precursors (pre-miRNA). These pre-miRNAs consist of an imperfect stem-loop structure. Pre-miRNAs are then exported from the nucleus into the cytoplasm by Exportin 5. In the cytoplasm, the hairpin precursors are cleaved by Dicer and its binding partner the transactivator RNA-binding protein TRBP into a small, imperfect dsRNA duplex (miRNA : miRNA*) that contains both the mature miRNA strand and its complementary strand. The miRNA strand is incorporated into the miRNP complex and targets complementary mRNA sequences, exerting its functionality via mRNA cleavage or translational repression.

 Mi(cro)RNAs are a contemporary class of tiny noncoding endogenous RNA molecules, only 18–25 nucleotides long. Since their discovery in 1993, these small molecules have been shown to play critical regulatory roles in a wide range of biological and pathological processes. Elucidating their mechanisms of action is still in its infancy. Nonetheless, work in this area to date has demonstrated that miRNAs may regulate cellular gene expression at the transcriptional or posttranscriptional level; by suppressing translation of protein coding genes, or cleaving target mRNAs to induce their degradation, through imperfect pairing with target mRNAs of protein coding genes [3]. MiRNA biogenesis in the human cell is a multistep complex process. A simplified representation is shown in Figure 1 [4]. The specific region of miRNA importance for mRNA target recognition is located at the 5′ end of the mature miRNA sequence, from bases 2 to 8. This is often referred to as the “seed sequence” [5]. Computational target prediction algorithms have been developed to identify putative mRNA targets, and these place considerable importance on this seed sequence, using it to search for complementary sequences in the 3′-UTRs of known genes that exhibit conservation across species. These algorithms predict that each miRNA may potentially bind to as many as 200 targets and estimate that miRNAs control the expression of at least one third of human mRNAs, further highlighting their crucial role as regulators of gene expression [6].

At the time of writing, 8 273 mature miRNA sequences have been described in primates, rodents, birds, fish, worms, flies, plants, and viruses [7]. This represents a growth of over 200 microRNAs in the last 2 years. In the human genome, over 600 mature miRNAs have been reported to date; however, computational prediction estimates that this could increase to >1000 [8]. It is obvious that the microRNA story is just beginning. 

## 2. Experimental Techniques for miRNA Analysis

The explosion of interest in miRNAs over the past two years necessitates effective tools for detecting their presence, quantification, and functional analysis. High-throughput profiling techniques such as miRNA microarrays and bead-based miRNA profiling have facilitated miRNA expression profiling, that is, far superior to existing low through-put techniques such as Northern blotting and cloning, and is essential for validation of microarray data. Castoldi et al. [9] described a novel miRNA microarray platform using locked nucleic acid-modified capture probes. Locked nucleic acid modification improved probe thermostability and increased specificity, thus enabling miRNAs with single nucleotide differences to be discriminated—an important consideration as sequence-related family members may be involved in different physiologic functions [10]. An alternative high-throughput miRNA profiling technique is the bead-based flow cytometric approach developed by Lu et al. [11]; a method which offers high specificity for closely related miRNAs because hybridization occurs in solution. Quantitative real-time PCR methodologies have been widely applied to miRNA research. To date, the most successful approach in terms of specificity and sensitivity is a two-step approach using looped miRNA-specific reverse transcription primers and TaqMan probes from Applied Biosystems [12]. 

To complement these miRNA profiling assays and to address functional questions necessitated the development of methods to manipulate miRNA expression. 2-*O*-Methyl antisense single-strand oligonucleotides and locked nucleic acid-modified oligonucleotides have been developed as miRNA inhibitors, making the suppression of endogenous miRNA activity and its downstream effect on mRNA expression achievable both in vitro and in vivo [13–16]. The effects of target miRNA knockdown on cell morphology and function can be determined using standard assays for processes such as cell proliferation, migration, invasion, and angiogenesis. MiRNA inhibition can be studied in animal models via transfection with tumor cells treated with miRNA inhibitors [17] or by the intravenous injection of “antagomirs” (2-*O*-methyl-modified nucleotides with a cholesterol moiety at the 3′-end [18]. The most recent development in the field of miRNA inhibition, led by Naldini and colleagues, describes techniques to manipulate miRNA expression in vivo by expressing decoy miRNA targets via lentiviral vectors [19]. This new approach to examine loss-of-function in vivo complements the results obtained by classic knockout technology as described above. It allows inhibition of specific miRNAs by building in multiple different decoys in the same miRNA inhibitor. This exciting new development should lead to answers for interesting functional questions with clinical or therapeutic relevance. For example, one could now potentially knock down the oncogenic proprieties of the miR-17-92-1 cluster which is well documented to be involved in human cancer [20]. This technique could also help one examine the let-7 microRNA family—a large, well-known tumor suppressor miRNA family [21] thereby providing insights into the functional consequence of knocking down all let-7 miRNAs [22].

MiRNA mimicry, a complementary technique to the aforementioned miRNA inhibition, has recently been used in vitro to identify the cellular processes and phenotypic changes associated with specific miRNAs transfected into cell lines [23]. Functional assays (e.g., proliferation, migration, invasion, and angiogenesis) then allow us to determine the effect of miRNA upregulation on tumorigenic or nontumorigenic cell populations. These revolutionary technologies will undoubtedly help us shed light on the functional roles of miRNAs and hold immense potential for application to the clinical arena as novel therapeutic targets.

### 2.1. MiRNA and Human Cancer

Early experimental work into the regulatory role of miRNAs uncovered their important role in various cellular processes such as differentiation, cell growth, and cell death. These processes are commonly dysregulated in cancer, implicating miRNAs in carcinogenesis. The first evidence of involvement of miRNAs in malignancy came from the identification of a translocation-induced deletion at chromosome 13q14.3 in B-cell chronic lymphocytic leukemia [24]. Loss of miR-15a and miR-16-1 from this locus results in increased expression of the antiapoptotic gene BCL2. Intensifying research in this field, using a range of techniques including miRNA cloning, quantitative PCR, microarrays and bead-based flow cytometric miRNA expression profiling has resulted in the identification and confirmation of abnormal miRNA expression in a number of human malignancies including breast cancer (Table 1). MiRNA expression has been observed to be upregulated or downregulated in tumours compared with normal tissue, supporting their dual role in carcinogenesis as either “Oncomirs” or tumour suppressors respectively [11].

The ability to obtain miRNA expression profiles from human tumors has led to remarkable insight and knowledge regarding the developmental lineage and differentiation states of tumours. Even within a single developmental lineage it has been shown that distinct patterns of miRNA expression are observed, that reflect mechanisms of transformation, and further support the idea that miRNA expression patterns encode the developmental history of human cancers. In contrast to messenger RNA (mRNA) profiles it is possible also to successfully classify poorly differentiated tumours using these new miRNA expression profiles [24, 25]. This has exciting implications clinically, in that miRNA expression may accurately diagnose poorly differentiated tissue samples which proved to be of uncertain histological origin thus facilitating treatment planning. Again in contrast to mRNA, Lu et al. showed that even a modest number of miRNAs are sufficient to classify human tumours and miRNAs remain largely intact in routinely collected, formalin-fixed, and paraffin-embedded clinical tissues [11]. Such information would eliminate the diagnostic uncertainty that previously existed in this setting and will be particularly useful for metastatic lesions of uncertain primary origin. 

### 2.2. Breast Cancer and Genomic Signatures

Recent advances in phenotyping and molecular profiling of human cancers have greatly enhanced the diagnosis and biological classification of several tumors, in particular breast cancers where this technology has enhanced disease classification beyond single-gene markers. Prior to this a very limited armamentarium of prognostic markers beyond those offered by histopathological analysis was available in the clinical arena. Pioneering work by Sorlie et al. [33–35] identified microarray-generated gene expression signatures which stratified breast cancers into intrinsic subtypes largely based on their ER, progesterone (PR), and HER2/*neu * receptor status. Subtypes were designated *Luminal A*, which strongly expressed ER and/or PR, but were HER2/*neu * negative; *Luminal B*, which were ER, PR, and HER2/*neu * (triple) positive; *Basal * tumours which were ER, PR, and HER2/*neu * triple negative; and an *HER2 * subset which was ER negative ER but had high expression of several genes in the HER2/*neu * amplicon, including *HER2 * and *GRB7*. Survival analyses showed significantly different outcome for patients depending on their tumour subtype, emphasising the clinical relevance of stratification by such molecular profiling. This novel method of disease stratification based on the molecular taxonomy of the breast tumour heralds the promise of improving and individualizing patients' treatment regimens [36]. Great scientific endeavours in this field of microarray-based gene expression profiling are ongoing and intensifying, with the aim of translating such technical advances to the clinical arena, in providing us with a new tool for accurate molecular diagnosis of breast cancer [37]. One such application recently has been the development by Paik et al. of a multigene assay predictive of recurrence of tamoxifen-treated, node-negative breast cancer (*Oncotype DX*) [38]. This, and other similarly novel genomic tests (e.g., *MammaPrint, Theros, MapQuant Dx*) prove the feasibility of accelerating the transition between empirical and molecular medicine. Analogous to the derivation of intrinsic subtypes of breast cancer from gene expression signatures, it is predicted that in the very near future miRNA signatures, which are currently showing capability of accurately classifying tumours according to currently available prognostic variables, will serve as novel biomarkers and prognostic indicators thus providing strong rationale for individualised treatment. Additionally it is thought that miRNAs have the potential to improve greatly the precision of the recently derived genomic signatures, given that miRNA profiles have superior accuracy to mRNA profiling in this regard [11]. A comprehensive interrogation of the breast cancer subclasses via miRNA expression profiling could further characterize the molecular basis underlying these subtypes, perhaps define more precise subsets of breast cancer, and provide opportunities for the identification of novel targets that can be exploited for targeted therapy.

### 2.3. MiRNA and Breast Cancer

Elucidation of the molecular mechanisms involved in breast cancer has been the subject of extensive research in recent years, yet several dilemmas and major challenges still prevail in the management of breast cancer patients including unpredictable response and development of resistance to adjuvant therapies. The emergence of miRNAs as regulators of gene expression identifies them as obvious novel candidate diagnostic and prognostic indicators, and potential therapeutic targets. Calin et al. [24] showed that half of the known mature human miRNAs are located in cancer-associated genomic regions, or fragile sites, thus potentiating their role in cancer. A specific example of this is the polycistron cluster miR-17-92 at the c13orf25 locus on chromosome 13q31. This locus is known to undergo loss of heterozygosity in a number of different cancer types, including breast cancer [39]. A number of other miRNAs (miR-196 and miR-10a) are located in homeobox clusters, which are known to be involved in the development of breast cancer and associated with the malignant capacity of cancer cells [40].

MiRNA expression studies in breast cancer indicate their importance and potential use as disease classifiers and prognostic tools in this field. In their analysis of 76 breast tumour and 34 normal specimens, Iorio et al. [26] identified 29 miRNAs that were differentially expressed in breast cancer tissue compared to normal, and a further set of 15 miRNAs that could correctly discriminate between tumour and normal. In addition, miRNA expression correlated with biopathological features such as ER and PR expression (*miR-30*) and tumour stage (*miR-213 * and *miR-203*). The differential expression of several *let-7 * isoforms was associated with biopathologic features including PR status (*let-7c*), lymph node metastasis (*let-7f-1, let-7a-3, let-7a-2*), or high proliferation index (*let-7c, let-7d*) in tumour samples. Mattie et al. identified unique sets of miRNAs associated with breast cancers currently defined by their HER2/*neu * or ER/PR status [27]*. * Significantly, there was overlap between the miRNAs identified in both studies. In initial studies in our own Department, we have shown that the expression levels of miR-195 and mir-154 are negatively correlated with ER positivity in a cohort of early breast cancers [41]. In another recent publication we were the first to identify reliable endogenous controls for analysis of miRNA by RQ-PCR in human breast tissue [42], subsequent to our validation of a two-gene normaliser (MRPL19 and PPIA) for analysis of gene expression in primary breast tissue [43].

## 3. Circulating microRNAs: Novel Minimally Invasive Biomarkers for Breast Cancer?

Current challenges in the management of breast cancer include a continuing search for sensitive minimally invasive markers that can be exploited to detect early neoplastic changes thus facilitating the detection of breast cancer at an early stage, as well as for monitoring the progress of patients with breast cancer and their response to treatments. Existing biomarkers for breast cancer have many inherent deficiencies. Mammography is currently the gold standard diagnostic tool however it is not without limitations, including its use of ionizing radiation and a false positive rate of 8–10% [44]*. * To date, only two markers have been established so far in the routine assessment of breast cancer: ER (for predicting response to endocrine therapies) and HER2 (for predicting response to Trastuzumab) [45]*. * Although these markers are currently available, ER and HER2 assessment is far from perfect [46]*. * A number of circulating tumour markers (e.g., carcinoembryonic antigen [CEA] and carbohydrate antigen 15-3 [CA 15-3]) are used clinically in the management of breast cancer, but the sensitivity of these markers is low, so that they are not useful as screening tools [47] though they have long been in clinical use as prognostic markers and to monitor for disease progression or recurrence. Despite their frequent use, CEA and Ca 15.3 remain poor markers for early stage disease with a documented preoperative sensitivity of only 9.11 and 5.36, respectively, as documented by Uehara et al. [48, 49]*.*

 The ideal biomarker should be easily accessible such that it can be sampled relatively noninvasively, sensitive enough to detect early presence of tumours in almost all patients and absent or minimal in healthy tumour-free individuals.

There is also great need for the identification of sensitive, reliable and acceptable markers of response to neoadjuvant and adjuvant therapies. MiRNAs have enormous potential to serve as an idea class of cancer biomarkers for the following reasons.

1. MiRNA expression is known to be aberrant in cancer [11, 24].
2. MiRNA expression profiles are pathognomonic, or tissue-specific [11].
3. MiRNAs are remarkably stable molecules that have been shown to be well preserved in formalin fixed, paraffin embedded tissues as well as fresh snap frozen specimens [50, 51].

Acknowledging the exceptional stability of miRNAs in visceral tissue very recently instigated efforts to establish if miRNAs were also preserved, detectable, and quantifiable in the circulation and other bodily fluids (urine, saliva, etc.). This area of miRNA research is only now emerging, and is generating much excitement in clinical and scientific communities, such as its potential. MiRNA presence in serum was described for the first time in March 2008, in patients with diffuse large B-cell lymphoma [52]. Subsequent to this, a small number of studies have reported similarly, on the presence of miRNA in circulation and their potential for use as novel biomarkers for diseases and physiological states including malignancy, diabetes mellitus and pregnancy [53–55]. However these studies have been limited by small numbers and inconsistencies in methodologies [56]. This concept needs extensive investigation to validate the theory. To date no work has been published on the role of circulating miRNAs in breast cancer—an area where, if feasible, their use as novel minimally invasive biomarkers would be an incredible breakthrough in our management of this disease.

## 4. Therapeutic Potential

The association of aberrant miRNA expression with tumorigenesis and the functional analysis of specific miRNAs illustrate the feasibility of using miRNAs as targets of therapeutic intervention. Anti-miRNA 2-*O*-methyl or locked nucleic acid oligonucleotides used to inactivate oncomirs such as miR-21 in breast tumors may taper tumor growth [17]. Anti-miR-21-induced reduction in tumor growth, interestingly, was also shown by Si et al. to be potentiated by the addition of the chemotherapeutic agent topotecan, an inhibitor of DNA topoisomerase I. This suggests that suppression of the oncogenic miR-21 could sensitize tumor cells to anticancer therapy, which is an exciting prospect for patients exhibiting a poor response to primary chemotherapy. Conversely, the induction of tumor suppressor miRNA expression using viral or liposomal delivery of tissue-specific tumor suppressors to affected tissue may result in the prevention of progression, or even shrinking, of breast tumors. Tumor suppressor miRNA induction has also been shown to be subject to epigenetic control. Using chromatin remodelling drugs to simultaneously inhibit DNA methylation and histone deacetylation, epigenetic alterations in cancer and normal cells were manipulated by Saito et al. [57], who showed that certain miRNAs were upregulated in tumor cells but not in normal cells. MiR-127, which exhibited reduced expression in 75% of human cancer cells tested, was significantly upregulated after treatment. The induction of this miRNA was associated with downregulation of the proto-oncogene BCL6, suggesting a cancer-protective effect for miR-127 and a novel therapeutic strategy for the prevention and treatment of malignancy. This concept of inducing tumour suppressor miRNA expression has been termed “miRNA Replacement Therapy”; in anticipation of the promising clinical potential it holds.

## 5. Conclusion

The involvement of miRNAs in the initiation and progression of human malignancy holds great potential for new developments in current diagnostic and therapeutic strategies in the management of patients with breast cancer. Much of the work on microRNAs is still in its infancy and requires further exploration so that we may better understand their role in tumorigenesis. This scientific endeavour will undoubtedly lead to exciting developments in the future management of breast cancer. As the functional roles of miRNAs in cancer biology are further uncovered we predict that; circulating miRNAs will serve as novel minimally invasive biomarkers for breast and other cancers, that improved methods of stratifying and subclassifying breast cancers will lead to tailored and individualized therapeutic regimens, thus sparing many patients from toxic effects of treatments from which they would derive no benefit. There is obviously great demand now for further intensive research into the identification of novel miRNAs, the elucidation of their mRNA targets, and an understanding of their functional effects, so as to improve our knowledge of the roles of these novel biomarkers in carcinogenesis and to expose their true potential as therapeutic agents.

## Figures and Tables

**Table 1:** MiRNAs with altered expression in malignancy.

Tissue/tumor type	Increased expression	Decreased expression
Breast [26, 27]	miR-21, miR-29b-2	miR-125b, miR-145 miR-10b, miR-155, miR-17-5p, miR-27b
Ovarian [28, 29]	miR-141, miR-200(a-c), miR-221	let-7f, miR-140, miR-145, miR199a, miR-424
Endometrial [30–32]	miR-103, miR-107, miR-185, miR-205, miR-210, miR-449	miR-99b, miR-152, miR-193, miR-204, miR-221, let-7i
Glioblastoma [4, 25]	miR-221, miR-21	miR-181a, miR-181b, miR-181c
Chronic lymphocytic leukaemia [24]		miR-15, miR-16
Lymphoma [4, 11]	miR-155, miR-17-92cluster	miR-15a
Colorectal [4, 11, 25]	miR-10a, miR-17-92 cluster, miR-20a, miR-24-1, miR-29b-2, miR-31	miR-143, miR-145, let-7
Thyroid [4, 25]	miR-221, miR-222, miR-146, miR-181b, miR-197, miR-346	
Hepatocellular [4, 25]	miR-18, miR-224	miR-199a, miR-195, miR-200a, miR-125a
Testicular [11]	miR-372, miR-373	
Pancreatic [4, 11, 25]	miR-221, miR-376a, miR301, miR-21, miR-24-2, miR-100, miR-103-1,2, miR-107, miR-125b-1	miR-375
Cholangiocarcinoma [25]	miR-21, miR-141, miR-200b	
Prostate [11]	let-7d, miR-195, miR-203	miR-128a
Gastric [4, 11, 25]	miR-223, miR-21, miR-103-2	miR-218-2
Lung [4, 11, 25]	mir-17-92 cluster, miR-17-5p	let-7 family

## References

[1] Garcia M (2007). *Global Cancer Facts & Figures 2007*.

[3] Jackson RJ, Standart N (2007). How do microRNA's regulate gene expression?. *Science's STKE*.

[4] Lowery AJ, Miller N, McNeill RE, Kerin MJ (2008). MicroRNAs as prognostic indicators and therapeutic targets: potential effect on breast cancer management. *Clinical Cancer Research*.

[5] Bartel DP (2004). MicroRNAs: genomics, biogenesis, mechanism, and function. *Cell*.

[6] Lewis BP, Burge CB, Bartel DP (2005). Conserved seed pairing, often flanked by adenosines, indicates that thousands of human genes are microRNA targets. *Cell*.

[7] Griffiths-Jones S, Saini HK, van Dongen S, Enright AJ (2008). miRBase: tools for microRNA genomics. *Nucleic Acids Research*.

[8] Berezikov E, Guryev V, van de Belt J (2005). Phylogenetic shadowing and computational identification of human microRNA genes. *Cell*.

[9] Castoldi M, Schmidt S, Benes V (2006). A sensitive array for microRNA expression profiling (miChip) based on locked nucleic acids (LNA). *RNA*.

[10] Abbott AL, Alvarez-Saavedra E, Miska EA (2005). The *let-7* MicroRNA family members *mir-48*, *mir-84*, and *mir-241* function together to regulate developmental timing in *Caenorhabditis elegans*. *Developmental Cell*.

[11] Lu J, Getz G, Miska EA (2005). MicroRNA expression profiles classify human cancers. *Nature*.

[12] Lao K, Xu NL, Yeung V, Chen C, Livak KJ, Straus NA (2006). Multiplexing RT-PCR for the detection of multiple miRNA species in small samples. *Biochemical and Biophysical Research Communications*.

[13] Hutvágner G, Simard MJ, Mello CC, Zamore PD (2004). Sequence-specific inhibition of small RNA function. *PLoS Biology*.

[14] Meister G, Landthaler M, Dorsett Y, Tuschl T (2004). Sequence-specific inhibition of microRNA-and siRNA-induced RNA silencing. *RNA*.

[15] Boutla A, Delidakis C, Tabler M (2003). Developmental defects by antisense-mediated inactivation of micro-RNAs 2 and 13 in *Drosophila* and the identification of putative target genes. *Nucleic Acids Research*.

[16] Ørom UA, Kauppinen S, Lund AH (2006). LNA-modified oligonucleotides mediate specific inhibition of microRNA function. *Gene*.

[17] Si M-L, Zhu S, Wu H, Lu Z, Wu F, Mo Y-Y (2007). miR-21-mediated tumor growth. *Oncogene*.

[18] Krutzfeldt J, Rajewsky N, Braich R (2005). Silencing of microRNAs in vivo with ‘antagomirs’. *Nature*.

[19] Gentner B, Schira G, Giustacchini A (2009). Stable knockdown of microRNA in vivo by lentiviral vectors. *Nature Methods*.

[20] Mendell JT (2008). miRiad roles for the miR-17-92 cluster in development and disease. *Cell*.

[21] Johnson CD, Esquela-Kerscher A, Stefani G (2007). The let-7 microRNA represses cell proliferation pathways in human cells. *Cancer Research*.

[22] Medina PP, Slack FJ (2009). Inhibiting microRNA function in vivo. *Nature Methods*.

[23] Franco-Zorrilla JM, Valli A, Todesco M (2007). Target mimicry provides a new mechanism for regulation of microRNA activity. *Nature Genetics*.

[24] Calin GA, Dumitru CD, Shimizu M (2002). Frequent deletions and down-regulation of micro-RNA genes miR15 and miR16 at 13q14 in chronic lymphocytic leukemia. *Proceedings of the National Academy of Sciences of the United States of America*.

[25] Volinia S, Calin GA, Liu C-G (2006). A microRNA expression signature of human solid tumors defines cancer gene targets. *Proceedings of the National Academy of Sciences of the United States of America*.

[26] Iorio MV, Ferracin M, Liu C-G (2005). MicroRNA gene expression deregulation in human breast cancer. *Cancer Research*.

[27] Mattie MD, Benz CC, Bowers J (2006). Optimized high-throughput microRNA expression profiling provides novel biomarker assessment of clinical prostate and breast cancer biopsies. *Molecular Cancer*.

[28] Iorio MV, Visone R, Di Leva G (2007). MicroRNA signatures in human ovarian cancer. *Cancer Research*.

[29] Dahiya N, Sherman-Baust CA, Wang T-L (2008). MicroRNA expression and identification of putative miRNA targets in ovarian cancer. *PLoS One*.

[30] Wu W, Lin Z, Zhuang Z, Liang X (2009). Expression profile of mammalian MicroRNAS in endometrioid adenocarcinoma. *European Journal of Cancer Prevention*.

[31] Chung TKH, Cheung T-H, Huen N-Y (2009). Dysregulated microRNAs and their predicted targets associated with endometrioid endometrial adenocarcinoma in Hong Kong women. *International Journal of Cancer*.

[32] Boren T, Xiong Y, Hakam A (2008). MicroRNAs and their target messenger RNAs associated with endometrial carcinogenesis. *Gynecologic Oncology*.

[33] Perou CM, Sørile T, Eisen MB (2000). Molecular portraits of human breast tumours. *Nature*.

[34] Sørlie T, Perou CM, Tibshirani R (2001). Gene expression patterns of breast carcinomas distinguish tumor subclasses with clinical implications. *Proceedings of the National Academy of Sciences of the United States of America*.

[35] Sørlie T, Wang Y (2006). Distinct molecular mechanisms underlying clinically relevant subtypes of breast cancer: gene expression analyses across three different platforms. *BMC Genomics*.

[36] Van't Veer LJ, Dai H, van de Vijver MJ (2002). Gene expression profiling predicts clinical outcome of breast cancer. *Nature*.

[37] Sotiriou C, Pusztai L (2009). Gene-expression signatures in breast cancer. *The New England Journal of Medicine*.

[38] Paik S, Shak S, Tang G (2004). A multigene assay to predict recurrence of tamoxifen-treated, node-negative breast cancer. *The New England Journal of Medicine*.

[39] Negrini M, Rasio D, Hampton GM (1995). Definition and refinement of chromosome 11 regions of loss of heterozygosity in breast cancer: identification of a new region at 11q23.3. *Cancer Research*.

[40] Makiyama K, Hamada J, Takada M (2005). Aberrant expression of HOX genes in human invasive breast carcinoma. *Oncology Reports*.

[41] Lowery AJ (2007). Micro-RNA expression profiling in primary breast tumours. *European Journal of Cancer*.

[42] Davoren PA, McNeill RE, Lowery AJ, Kerin MJ, Miller N (2008). Identification of suitable endogenous control genes for microRNA gene expression analysis in human breast cancer. *BMC Molecular Biology*.

[43] McNeill RE, Miller N, Kerin MJ (2007). Evaluation and validation of candidate endogenous control genes for real-time quantitative PCR studies of breast cancer. *BMC Molecular Biology*.

[44] Taplin S, Abraham L, Barlow WE (2008). Mammography facility characteristics associated with interpretive accuracy of screening mammography. *Journal of the National Cancer Institute*.

[45] Thompson A, Brennan K, Cox A (2008). Evaluation of the current knowledge limitations in breast cancer research: a gap analysis. *Breast Cancer Research*.

[46] Piccart-Gebhart MJ, Procter M, Leyland-Jones B (2005). Trastuzumab after adjuvant chemotherapy in HER2-positive breast cancer. *The New England Journal of Medicine*.

[47] Harris L, Fritsche H, Mennel R (2007). American society of clinical oncology 2007 update of recommendations for the use of tumor markers in breast cancer. *Journal of Clinical Oncology*.

[48] O'Hanlon DM, Kerin MJ, Kent P, Maher D, Grimes H, Given HF (1995). An evaluation of preoperative CA 15-3 measurement in primary breast carcinoma. *British Journal of Cancer*.

[49] Uehara M, Kinoshita T, Hojo T, Akashi-Tanaka S, Iwamoto E, Fukutomi T (2008). Long-term prognostic study of carcinoembryonic antigen (CEA) and carbohydrate antigen 15-3 (CA 15-3) in breast cancer. *International Journal of Clinical Oncology*.

[50] Xi Y, Nakajima G, Gavin E (2007). Systematic analysis of microRNA expression of RNA extracted from fresh frozen and formalin-fixed paraffin-embedded samples. *RNA*.

[51] Li J, Smyth P, Flavin R (2007). Comparison of miRNA expression patterns using total RNA extracted from matched samples of formalin-fixed paraffin-embedded (FFPE) cells and snap frozen cells. *BMC Biotechnology*.

[52] Lawrie CH, Gal S, Dunlop HM (2008). Detection of elevated levels of tumour-associated microRNAs in serum of patients with diffuse large B-cell lymphoma. *British Journal of Haematology*.

[53] Mitchell PS, Parkin RK, Kroh EM (2008). Circulating microRNAs as stable blood-based markers for cancer detection. *Proceedings of the National Academy of Sciences of the United States of America*.

[54] Chen X, Ba Y, Ma L (2008). Characterization of microRNAs in serum: a novel class of biomarkers for diagnosis of cancer and other diseases. *Cell Research*.

[55] Gilad S, Meiri E, Yogev Y (2008). Serum microRNAs are promising novel biomarkers. *PLoS One*.

[56] Chin LJ, Slack FJ (2008). A truth serum for cancer—microRNAs have major potential as cancer biomarkers. *Cell Research*.

[57] Saito Y, Liang G, Egger G (2006). Specific activation of microRNA-127 with downregulation of the proto-oncogene BCL6 by chromatin-modifying drugs in human cancer cells. *Cancer Cell*.

